# Supplementary material for: A non-canonical function for Centromere-associated protein-E controls centrosome integrity and orientation of cell division
Source: Commun Biol. 2021 Mar 19;4:358. doi: 10.1038/s42003-021-01861-4 (PMC7979751; doi:10.1038/s42003-021-01861-4)
Supplement: Supplementary file 2 — Supplementary Information [file 42003_2021_1861_MOESM2_ESM.pdf]

Supplementary information

A non-canonical function for Centromere-associated protein-E controls centrosome integrity and orientation of cell division

Mikito Owa and Brian Dynlacht

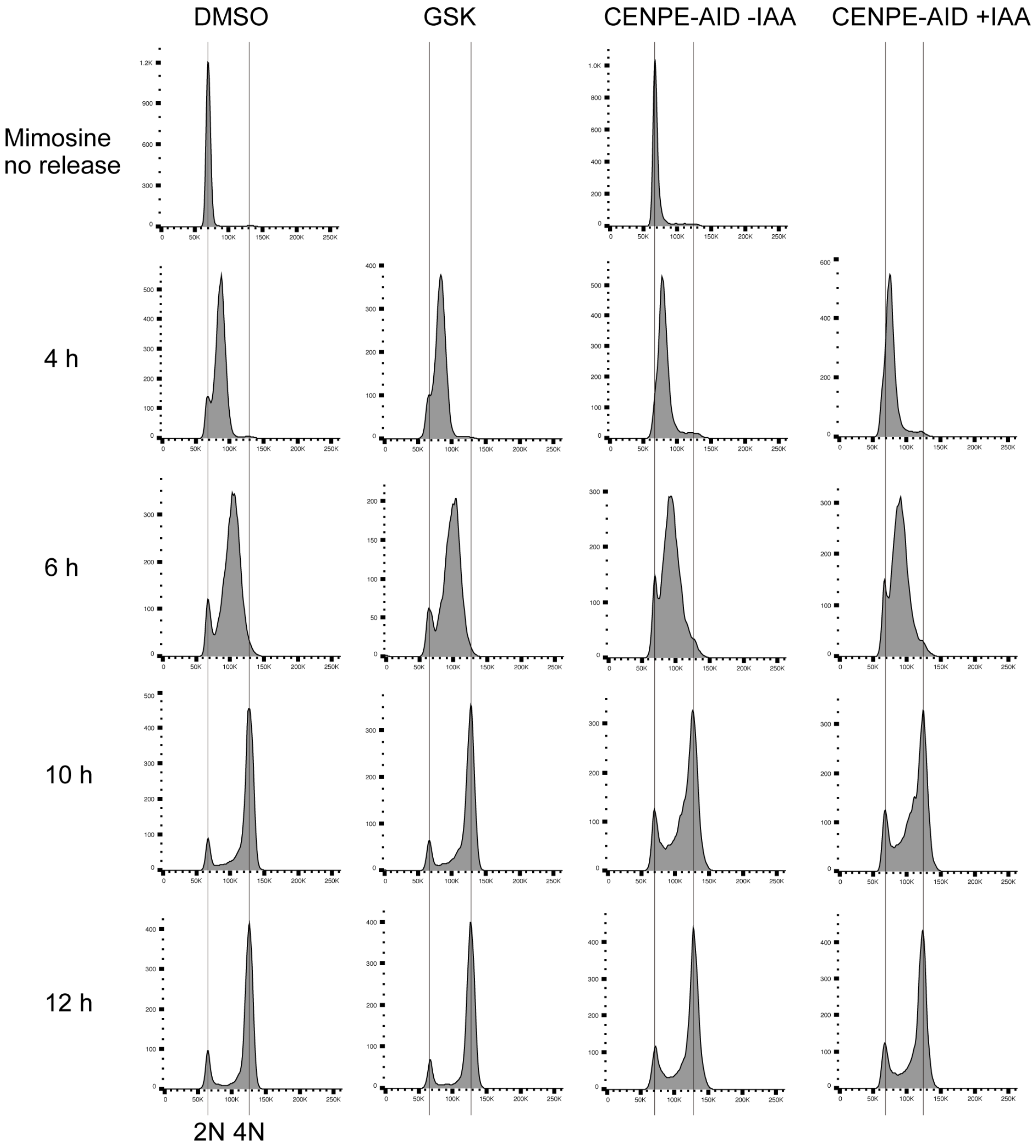

Supplementary Fig. 1. Cell cycle profiles of mimosine-synchronized cells.

Mimosine synchronized wild-type RPE-1 cells or CENP-E-AID cells treated with indicated drugs were released for 4, 6, 10 and 12 hours and analyzed by flow cytometry. Histograms of DNA content at each time point are shown (2N: G_1; 4N: G_2/M)

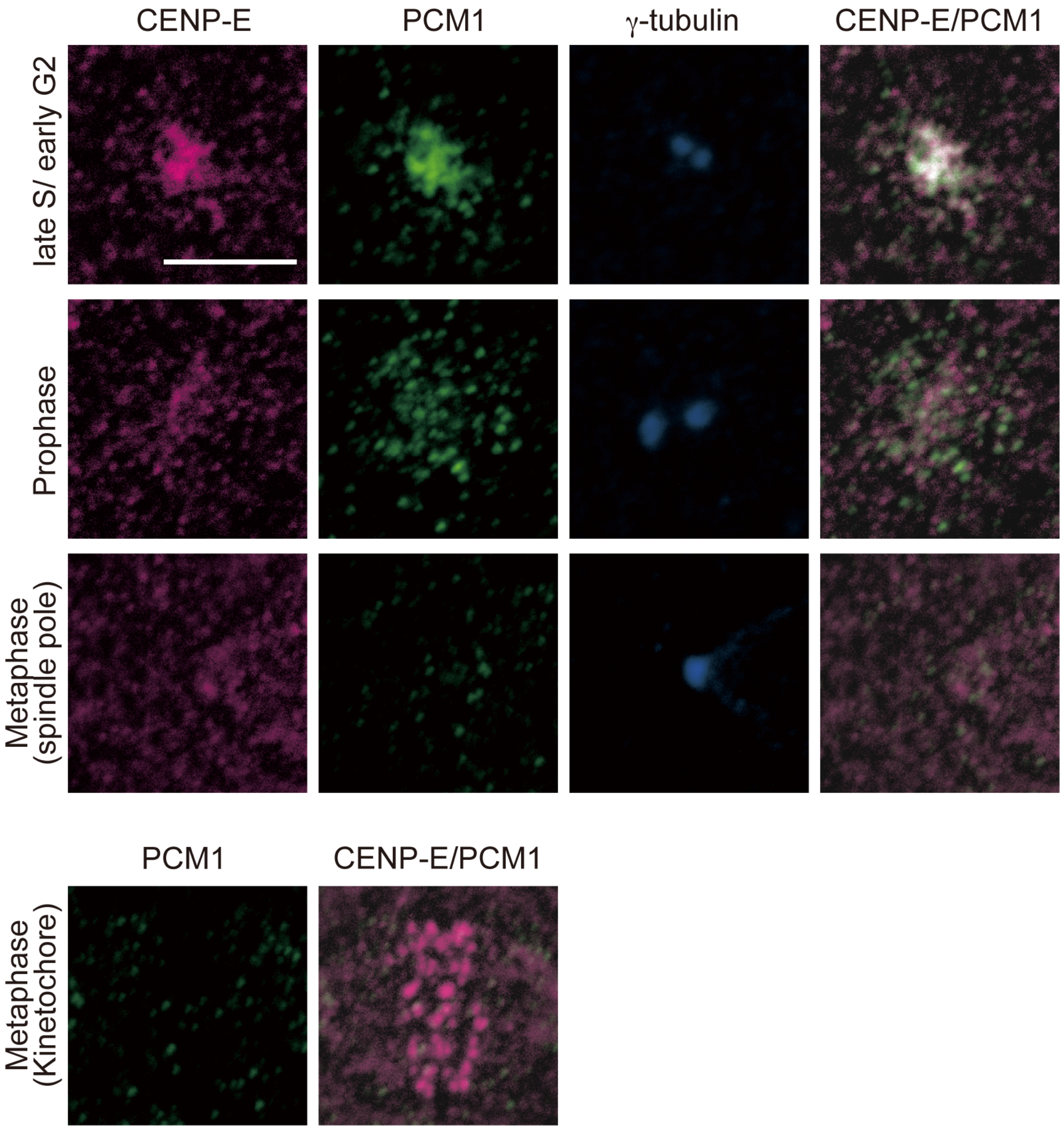

Supplementary Fig. 2. CENP-E co-localizes with PCM1 in late S/early G_2 phase.

Wild-type RPE-1 cells were co-stained with indicated antibodies. Representative images for z-stacks around centrosomes in late S/early G_2, prophase, or metaphase cells are shown. For metaphase cells, PCM1 and CENP-E/PCM1 merged images are also presented in the bottom-most panels. Scale bar=10 μm.

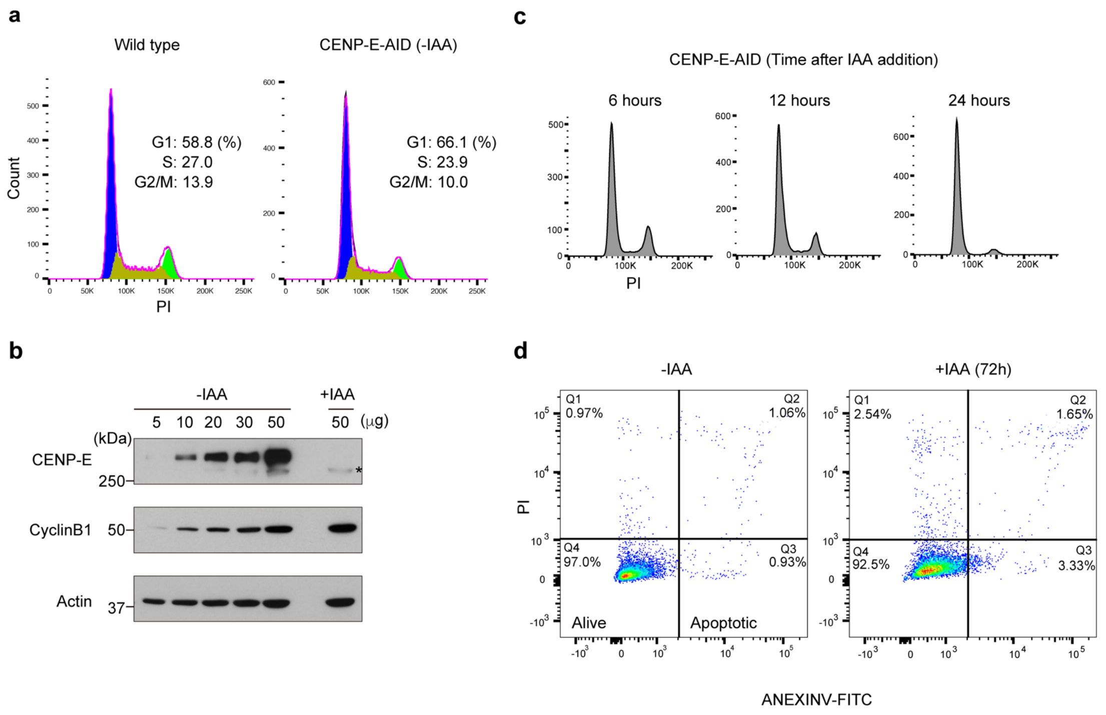

Supplementary Fig. 3. Further characterization of the CENP-E-AID cell line.

(a) Cell cycle profiles of asynchronous wild-type cells and asynchronous CENP-E-AID cells without IAA treatment. DNA content was analyzed by FACS using propidium iodide (PI) staining. **(b)** Western blots of serial dilutions of CENP-E lysates from mitotic CENP-E-AID cells with or without IAA (treated for 1 hour) were probed with indicated antibodies. *: non-specific band in long exposure. **(c)** Cell cycle profiles of CENP-E-AID cells treated with IAA. Asynchronous CENP-E-AID cells were treated with IAA for indicated hours and analyzed by FACS (PI). Time on the histograms indicates duration of IAA treatment. **(d)** Apoptosis in CENP-E KO cells was analyzed by FACS using AnnexinV-FITC staining.

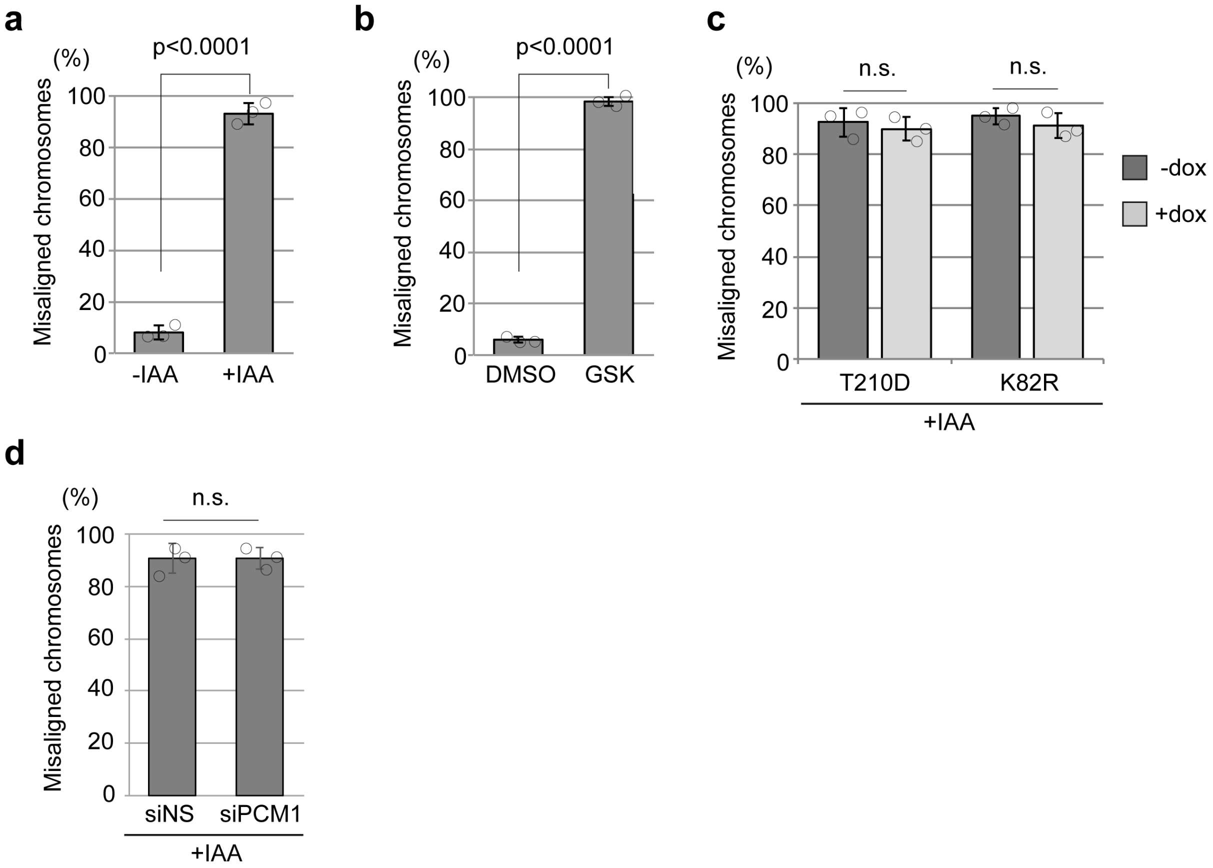

Supplementary Fig. 4. Percentages of misaligned chromosomes.

Percentages of cells that possess one or more misaligned chromosomes around the spindle pole (>100 cells in total from three independent experiments; error bars: SD). **(a)** CENP-E-AID cells with or without IAA; (**b)** wild-type cells treated with DMSO or GSK; (**c)** CENP-E-AID cells stably expressing doxycycline (dox)-inducible V5-tagged Plk1 (T210D or K82R), treated with IAA only or IAA and dox; (**d)** CENP-E-AID cells treated with control siRNA and IAA or siPCM1 and IAA.

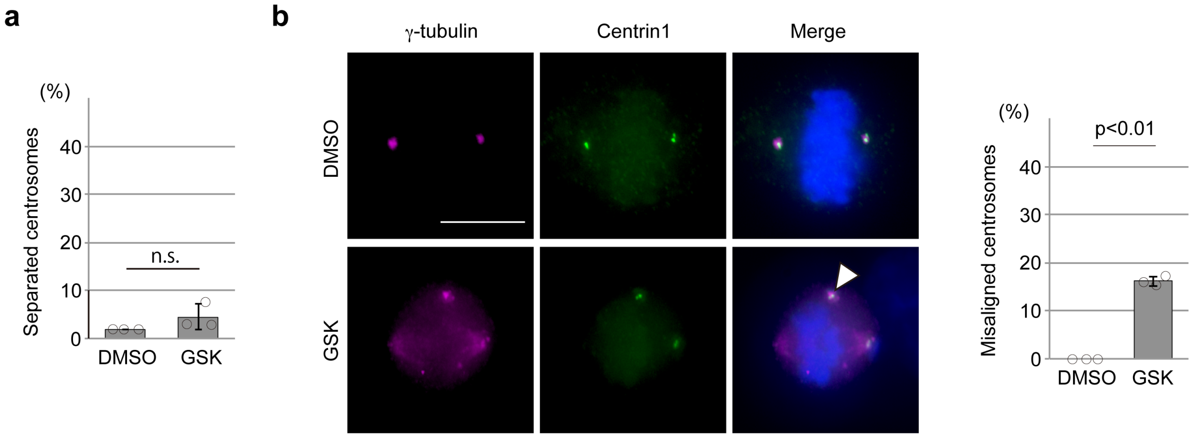

Supplementary Fig. 5. Centriole detachment from the spindle pole in CENP-E KO. Wild-type RPE-1 cells were synchronized with thymidine and released for 8 hours with DMSO or GSK. The cells were co-immuno-stained with antibodies against γ-tubulin (magenta) and centrin1 (green). **(a)** Percentages of precocious centriole disengagement (>100 cells in total from three independent experiments; error bars: SD). **(b)** Representative images for normal (top, DMSO) or misaligned centrioles (bottom, GSK). The arrowhead indicates centrioles detached from the spindle pole. Percentages of centrioles detached from the spindle pole are shown in the bar graph (>100 cells in total from three independent experiments; error bars: SD). p-values were calculated by unpaired *t*-tests. Scale bar=10 μm.

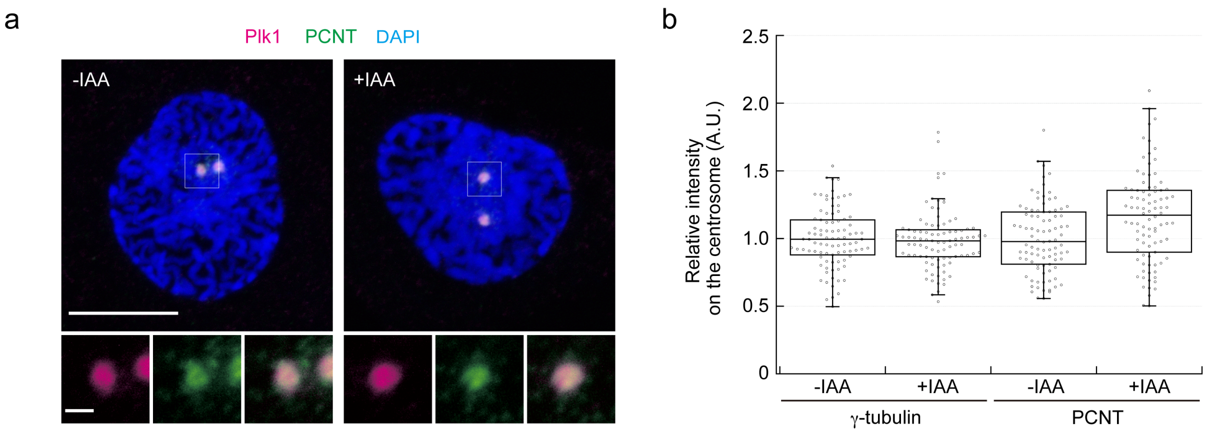

Supplementary Fig. 6. Centrosome maturation was not perturbed by loss of CENP-E.

(a, b) Representative images for IAA-treated or un-treated CENP-E-AID cells in prophase co-immunostained with antibodies against γ-tubulin (magenta) and PCNT (green) (**a**, Scale bar=10 μm). The area enclosed by the square in each image is magnified and shown under the panel (Scale bar=1 μm). Relative γ-tubulin or PCNT intensities on the centrosome in prophase cells were plotted in (**b**) (N=90, three independent experiments; whisker: 95% confidence interval; box: interquartile; center line: median).

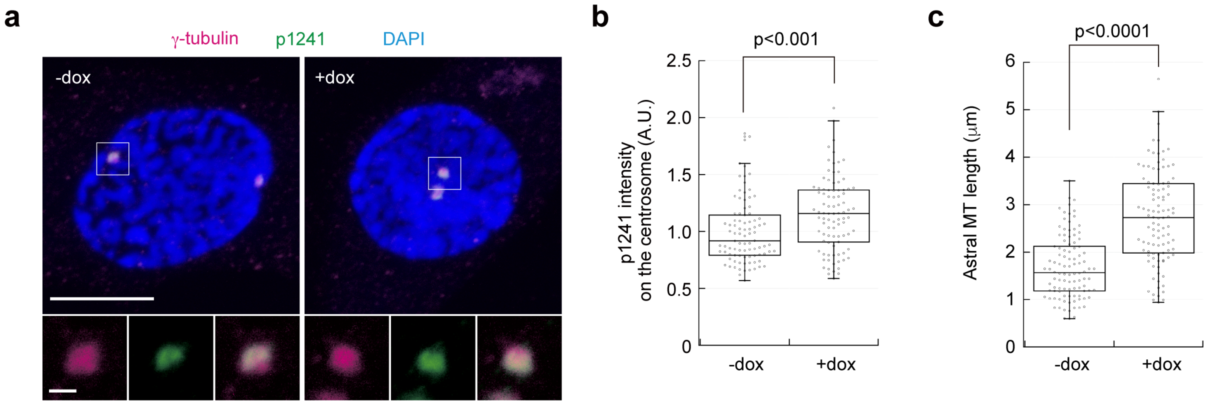

Supplementary Fig. 7. Phenotypes caused by loss of CENP-E are rescued by Plk1(T210D) overexpression. (**a, b**) Representative images for CENP-E KO cells expressing (+dox) or not expressing (-dox) Plk1 (T210D) in prophase co-immunostained with antibodies against γ-tubulin (magenta) and PCNT-pS1241 (green). Scale bar=10 μm. The area enclosed by the square in each image is magnified and shown under the panel (Scale bar=1 μm). In +dox samples, 1 μg/ml dox was maintained throughout the experiments. Relative PCNT-pS1241 intensities on the centrosome in prophase cells are plotted in (**b**) (N=90, three independent experiments; whisker: 95% confidence interval; box: interquartile; center line: median). **(c)** Length of astral MTs in mitotic CENP-E KO cells expressing (+dox) or not expressing (-dox) Plk1 (T210D) was measured and plotted (N=102, three independent experiments; whisker: 95% confidence interval; box: interquartile; center line: median). p-values were calculated by Mann-Whitney *U* tests.

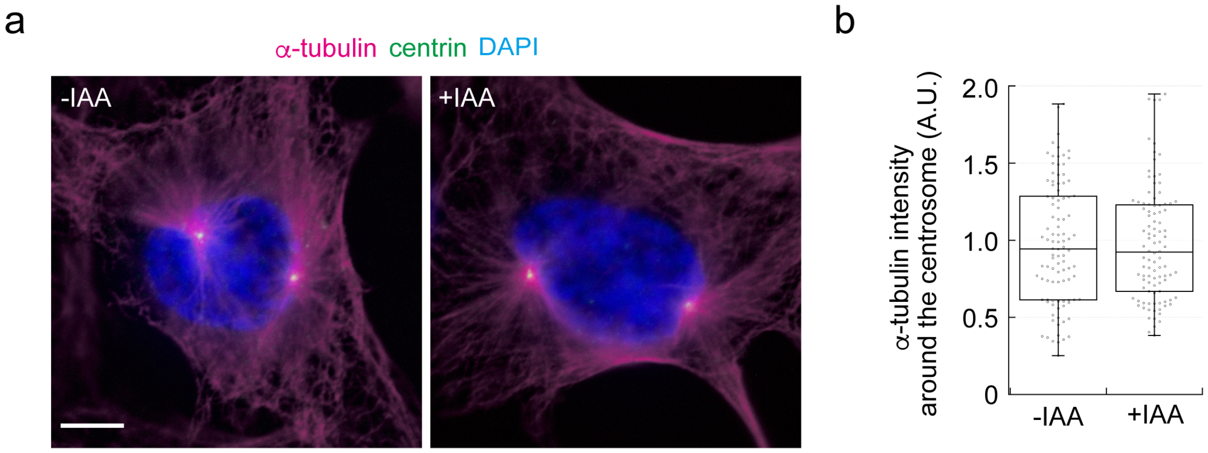

Supplementary Fig. 8. MT network in prophase was not affected by loss of CENP-E.

(a, b) Representative images for IAA-treated or un-treated CENP-E-AID cells in prophase co-immunostained with antibodies against α-tubulin (magenta) and centrin (green) (**a**, Scale bar=10 μm). Relative intensities around the centrosome in prophase cells were plotted in (**b**) (N=100, three independent experiments; whisker: 95% confidence interval; box: interquartile; center line: median).

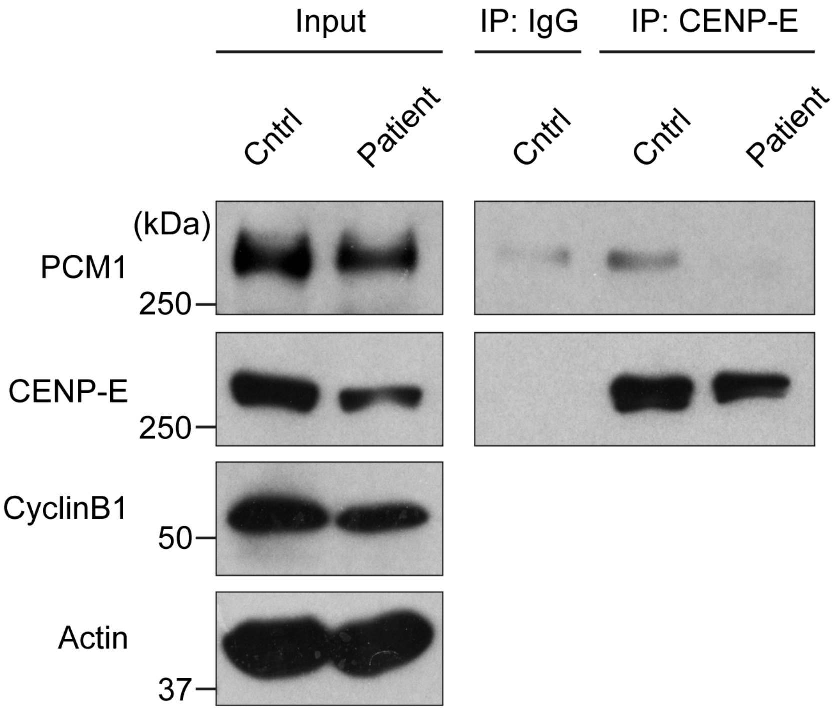

Supplementary Fig. 9. Immunoprecipitation of CENP-E in LCLs.

Control (Cntrl) or patient LCLs synchronized by single thymidine block were released into fresh medium for 5 hours. Cell lysates from those cells were immunoprecipitated with the CENP-E antibody. Inputs and eluates were blotted with indicated antibodies. For IP control, an anti-GFP antibody (mouse IgG) was used.

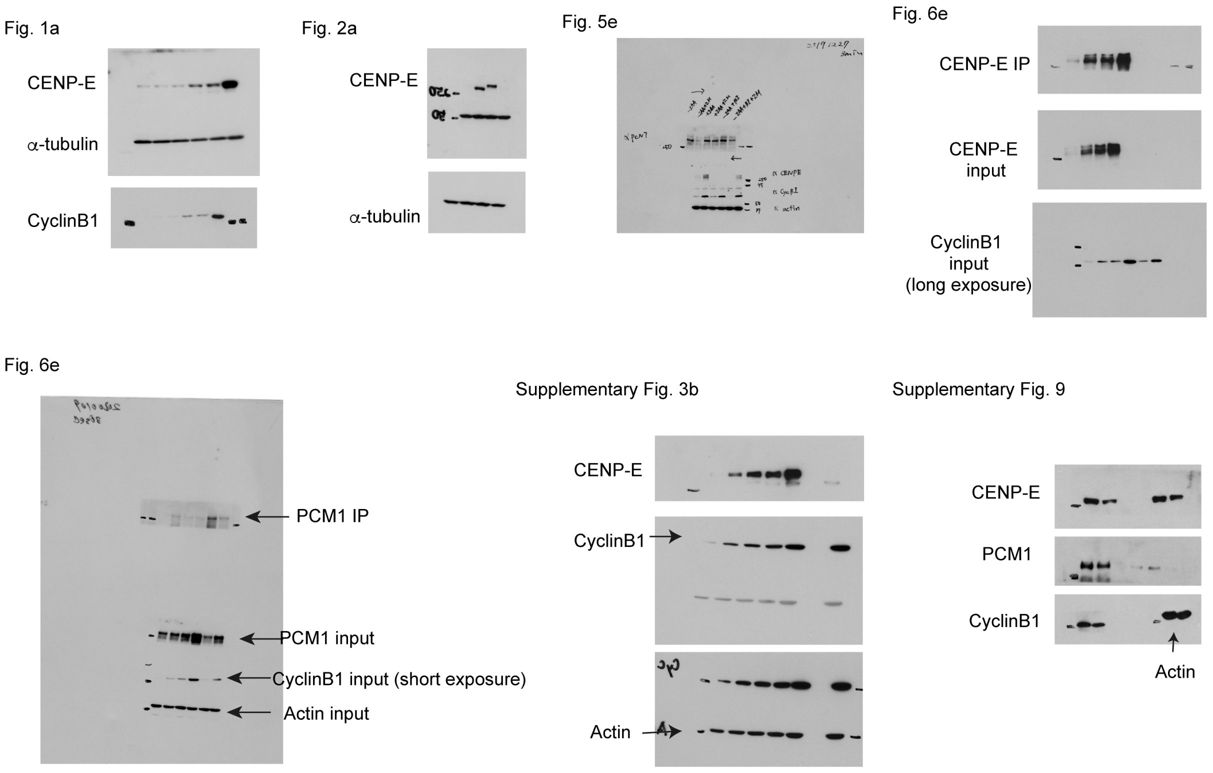

Supplementary Fig. 10. Uncropped images of western blots.

Supplementary Table 1: antibodies used in this study

Antibody	Host	Dilution	Reference
γ-tubulin	Mouse	1:2000 (IF)	Sigma #T5326
Centrin (20H5)	Mouse	1:1000 (IF)	Merc Millipore #04-1624
Centrin1	Rabbit	1:400 (IF)	Proteintech #12794-1-AP
Plk1 (F-8)	Mouse	1:200 (IF)	Santa Cruz #sc-376685
CENP-E (C-5)	Mouse	1:100 (IF), 1:500 (WB)	Santa Cruz #sc-17783
PCNT	Rabbit	1:1000 (IF)	Covance #PRB-432C
PCNT	Rabbit	1:20000 (IF), 1:5000 (WB)	Lee and Rhee, 2011
PCNT-p1241	Rabbit	1:400 (IF)	Lee and Rhee, 2011
α-tubulin (B5-1-2)	Mouse	1:3000 (IF)	Sigma #T5168
V5	Mouse	1:1000 (IF)	Invitrogen #R960-25
PCM1	Rabbit	1:1000 (IF), 1:500 (WB)	Santa Cruz #sc-67204
CyclinB1 (GNS1)	Mouse	1:1000 (WB)	Santa Cruz #sc-245
β-actin	Mouse	1:10000 (WB)	Abcam #ab6276
