# Supplementary material for: A non-canonical function for Centromere-associated protein-E controls centrosome integrity and orientation of cell division
Source: Commun Biol. 2021 Mar 19;4:358. doi: 10.1038/s42003-021-01861-4 (PMC7979751; doi:10.1038/s42003-021-01861-4)
Supplement: Supplementary file 3 — Description of Additional Supplementary Files [file 42003_2021_1861_MOESM3_ESM.pdf]

Description of Additional Supplementary Files

File name: Supplementary Movie 1

Description: Live cell imaging of H2B-GFP in CENP-E-AID cells with or without IAA.

File name: Supplementary Data 1

Description: Original data for graphs.
