# Supplementary material for: A non-canonical function for Centromere-associated protein-E controls centrosome integrity and orientation of cell division
Source: Commun Biol. 2021 Mar 19;4:358. doi: 10.1038/s42003-021-01861-4 (PMC7979751; doi:10.1038/s42003-021-01861-4)
Supplement: Supplementary file 6 — Reporting Summary [file 42003_2021_1861_MOESM6_ESM.pdf]

nature research

Corresponding author(s): Mikito Owa, Brian Dynlacht

Last updated by author(s): Jan 29, 2021

# Reporting Summary

Nature Research wishes to improve the reproducibility of the work that we publish. This form provides structure for consistency and transparency in reporting. For further information on Nature Research policies, see our Editorial Policies and the Editorial Policy Checklist.

## Statistics

For all statistical analyses, confirm that the following items are present in the figure legend, table legend, main text, or Methods section.

| n/a | Confirmed | |
|---|---|---|
| ☐ | ☒ | The exact sample size (*n*) for each experimental group/condition, given as a discrete number and unit of measurement |
| ☐ | ☒ | A statement on whether measurements were taken from distinct samples or whether the same sample was measured repeatedly |
| ☐ | ☒ | The statistical test(s) used AND whether they are one- or two-sided<br>*Only common tests should be described solely by name; describe more complex techniques in the Methods section.* |
| ☒ | ☐ | A description of all covariates tested |
| ☐ | ☒ | A description of any assumptions or corrections, such as tests of normality and adjustment for multiple comparisons |
| ☐ | ☒ | A full description of the statistical parameters including central tendency (e.g. means) or other basic estimates (e.g. regression coefficient) AND variation (e.g. standard deviation) or associated estimates of uncertainty (e.g. confidence intervals) |
| ☒ | ☐ | For null hypothesis testing, the test statistic (e.g. *F*, *t*, *r*) with confidence intervals, effect sizes, degrees of freedom and *P* value noted<br>*Give P values as exact values whenever suitable.* |
| ☒ | ☐ | For Bayesian analysis, information on the choice of priors and Markov chain Monte Carlo settings |
| ☒ | ☐ | For hierarchical and complex designs, identification of the appropriate level for tests and full reporting of outcomes |
| ☒ | ☐ | Estimates of effect sizes (e.g. Cohen's *d*, Pearson's *r*), indicating how they were calculated |

*Our web collection on statistics for biologists contains articles on many of the points above.*

## Software and code

Policy information about availability of computer code

| | |
|---|---|
| Data collection | Not applicable |
| Data analysis | Not applicable |

For manuscripts utilizing custom algorithms or software that are central to the research but not yet described in published literature, software must be made available to editors and reviewers. We strongly encourage code deposition in a community repository (e.g. GitHub). See the Nature Research guidelines for submitting code & software for further information.

## Data

Policy information about availability of data

All manuscripts must include a data availability statement. This statement should provide the following information, where applicable:

- Accession codes, unique identifiers, or web links for publicly available datasets
- A list of figures that have associated raw data
- A description of any restrictions on data availability

All materials except LCLs and data are available from corresponding authors upon reasonable requests. LCLs were obtained from Dr. G. Mirzaa (Seattle Children's Institute), and we are not permitted to give them to a third party ourselves. The patients consented to using the cell lines for research.

# Field-specific reporting

# Life sciences study design

All studies must disclose on these points even when the disclosure is negative.

| | |
|---|---|
| Sample size | Sample size was determined based on previous studies which performed similar experiments. |
| Data exclusions | No data were excluded. |
| Replication | All experiments were performed at least two or three times. |
| Randomization | Not applicable |
| Blinding | Not applicable |

# Reporting for specific materials, systems and methods

We require information from authors about some types of materials, experimental systems and methods used in many studies. Here, indicate whether each material, system or method listed is relevant to your study. If you are not sure if a list item applies to your research, read the appropriate section before selecting a response.

## Materials & experimental systems

| n/a | Involved in the study | |
|---|---|---|
| ☐ | ☒ | Antibodies |
| ☐ | ☒ | Eukaryotic cell lines |
| ☒ | ☐ | Palaeontology and archaeology |
| ☒ | ☐ | Animals and other organisms |
| ☒ | ☐ | Human research participants |
| ☒ | ☐ | Clinical data |
| ☒ | ☐ | Dual use research of concern |

## Methods

| n/a | Involved in the study | |
|---|---|---|
| ☒ | ☐ | ChIP-seq |
| ☐ | ☒ | Flow cytometry |
| ☒ | ☐ | MRI-based neuroimaging |

## Antibodies

| | |
|---|---|
| Antibodies used | Described in Supplementary Table1 |
| Validation | Catalog numbers of the antibodies or references are described in Supplementary Table1 |

## Eukaryotic cell lines

Policy information about cell lines

| | |
|---|---|
| Cell line source(s) | hTERT-RPE-1 obtained from ATCC; Lymphoblastoid cell lines obtained from G. Mirzaa |
| Authentication | hTERT-RPE-1 were obtained from ATCC and have been authenticated by ATCC. |
| Mycoplasma contamination | Mycoplasma contamination was tested and all cell lines were negative. |
| Commonly misidentified lines (See ICLAC register) | Not applicable |

## Flow Cytometry

### Plots

Confirm that:

☒ The axis labels state the marker and fluorochrome used (e.g. CD4-FITC).

☒ The axis scales are clearly visible. Include numbers along axes only for bottom left plot of group (a 'group' is an analysis of identical markers).

☒ All plots are contour plots with outliers or pseudocolor plots.

☒ A numerical value for number of cells or percentage (with statistics) is provided.

## Methodology

| | |
|---|---|
| Sample preparation | Described in methods. |
| Instrument | LSRII UV cell analyzer (BD bioscience) |
| Software | FlowJo |
| Cell population abundance | All analyses are from a homogeneous population (established breast cancer cell lines), so after sorting out doublets, the 85-99% of the cells remain for analysis, counting at least 5000 single cells. |
| Gating strategy | We selected our population first screening FSC/SCC. Then, to exclude doublets, we plotted FSC-H/FSC-A. Single cell population was selected for further analysis and plotted for PI/Ki67-FITC. |

☐ Tick this box to confirm that a figure exemplifying the gating strategy is provided in the Supplementary Information.
